# Mesenchymal stromal cells in cancer: a review of their immunomodulatory functions and dual effects on tumor progression

**DOI:** 10.1002/path.5357

**Published:** 2019-12-18

**Authors:** Sabine Galland, Ivan Stamenkovic

**Affiliations:** ^1^ Laboratory of Experimental Pathology Institute of Pathology, CHUV Lausanne Switzerland

**Keywords:** mesenchymal stem/stromal cells, immune system, inflammation, cancer, Toll‐like receptors, exosomes, anti‐tumor therapy, interleukin‐6

## Abstract

Mesenchymal stem or stromal cells (MSCs) are pluripotent cells implicated in a broad range of physiological events, including organogenesis and maintenance of tissue homeostasis as well as tissue regeneration and repair. Because their current definition is somewhat loose – based primarily on their ability to differentiate into a variety of mesenchymal tissues, adhere to plastic, and express, or lack, a handful of cell surface markers – MSCs likely encompass several subpopulations, which may have diverse properties. Their diversity may explain, at least in part, the pleiotropic functions that they display in different physiological and pathological settings. In the context of tissue injury, MSCs can respectively promote and attenuate inflammation during the early and late phases of tissue repair. They may thereby act as sensors of the inflammatory response and secrete mediators that boost or temper the response as required by the stage of the reparatory and regenerative process. MSCs are also implicated in regulating tumor development, in which they are increasingly recognized to play a complex role. Thus, MSCs can both promote and constrain tumor progression by directly affecting tumor cells via secreted mediators and cell–cell interactions and by modulating the innate and adaptive immune response. This review summarizes our current understanding of MSC involvement in tumor development and highlights the mechanistic underpinnings of their implication in tumor growth and progression. © 2020 Authors. *Journal of Pathology* published by John Wiley & Sons Ltd on behalf of Pathological Society of Great Britain and Ireland.

## Introduction

Few cells have attracted as much interest in the past 20 years as have mesenchymal stem or stromal cells (MSCs). The fascination with MSCs is due in part to their implication in a wide range of physiological and pathological processes, including development, tissue repair, organ transplantation, autoimmunity, and cancer, and in part to their elusive identity. There have been several excellent recent reviews on MSCs 1, 2, 3, 4, 5, 6, 7, 8, 9 and rather than discuss all of their known biological and functional properties, we will focus on their role in cancer, particularly their immunomodulatory ability.

Current experimental models suggest that MSCs may both promote and constrain tumor growth, although their net effect appears to be predominantly pro‐tumorigenic (Table 1, with references). Tumor growth, which triggers and maintains chronic inflammation, tissue remodeling, and dampened immunity, has been heralded as ‘a wound that never heals’ 44, in which MSCs actively participate. The immunomodulatory effects of MSCs on innate and adaptive immunity through secreted factors, exosomes, and cell–cell contacts constitute a major mechanism by which MSCs affect tumor initiation and progression. As MSCs may exert opposing effects on immune cells, promoting inflammation on the one hand and exhibiting immunosuppressive features, which favor tumor progression, on the other, harnessing their plasticity toward the expression of anti‐tumorigenic, anti‐inflammatory, and pro‐immunogenic properties may provide an attractive therapeutic option. However, such an endeavor requires in‐depth understanding of the functional relationship between tumor cells, MSCs, and immune cells – particularly how tumor cells subvert MSCs to function in their favor and the underpinnings of MSC plasticity that allow such subversion to occur.

**Table 1:** Pro‐ and anti‐tumor effects of MSCs in the TME

References	Origin of MSCs	Species	Tumor relevance	Tumor function	Mechanisms
10	Bone marrow	Human	Breast cancer	Promoting	CCL5 (RANTES)
11	Bone marrow	Human	Breast cancer	Promoting	p‐EGFR
12	Bone marrow	Human	Prostate cancer	Promoting	TGF‐β
13	Adipose tissue	Human	Prostate cancer	Promoting	TGF‐β and periostin
14	Umbilical cord and adipose tissue	Human	Breast cancer	Suppressive	Apoptosis induction (PARP cleavage)
15	Bone marrow	Mouse	Hepatoma	Suppressive	Apoptosis induction
16	Bone marrow	Rat and mouse	Melanoma	Suppressive when administered at a 3:1 ratio with ECs	Cytotoxic for endothelial cells (ROS) and anti‐angiogenic effects
17	Bone marrow	Human	Ovarian cancer (‘SKOV‐3’ cell line)	Promoting	IL‐6; transition to CAF
18	Umbilical cord blood (UCB) and adipose tissue (AT)	Human	Glioblastoma multiforme	UCB‐MSCs: suppressive; AT‐MSCs: promoting	UCB‐MSCs: TRAIL (apoptosis) AT‐MSCs: VEGF, ANG1, PDGF, IGF, SDF‐1/CXCL12
19	Tumor tissue, bone marrow	Human	Gastric cancer	Promoting	IL‐8
20	Tumor tissue, bone marrow	Human	Glioma	Promoting	IL‐6/STAT3
21	Bone marrow	Human	Breast cancer ‘MDA‐MB‐231’ cells	Promoting	CAF differentiation
22	Bone marrow	Human	Breast cancer ‘MDA‐MB‐231’ cells	Promoting	TGF‐β/Smad pathway; TGF‐β‐dependent transition to CAF
23	Bone marrow	Human	Kaposi's sarcoma	Suppressive	MSCs target Akt activity within tumor cells
24	Dermal tissues of a human fetus	Human	Hepatoma	Suppressive	Wnt signaling pathway
25	Tumor tissue	Human	Gastric cancer	Promoting	SDF‐1 and VEGF
26	Tumor tissue	Human	Head and neck cancers	Promoting	IL‐6, IL‐8, SDF‐1α, and expression of CD54
27	Bone marrow	Human	Inflammatory breast cancer	Promoting	IL‐6
28	Bone marrow	Human	Pancreatic cancer	Suppressive	Unknown
29	Bone marrow	Human	Glioma	Suppressive	Downregulation of PDGF/PDGFR axis
30	Tumor tissue	Human	Ovarian cancer	Promoting	Promotion of Akt and XIAP phosphorylation
31	Tumor tissue	Human	Colon cancer	Promoting	IL‐6/Notch‐1/CD44 signaling axis
32	Bone marrow	Mouse	Melanoma	Promoting	Immunosuppression after priming by IFN‐γ and TNF‐α
33	Tumor tissue	Human	Hepatocellular carcinoma	Promoting	Trophic factor secretion
34	Bone marrow	Human and rat	Colon cancer	Suppressive	Immunomodulation and decrease in inflammation; increase of miR‐150 and miR‐7
35	Bone marrow	Human	Gastric cancer	Promoting	Platelet activation: TGF‐β
36	Bone marrow	Mouse	Breast cancer	Promoting	Increased stiffness (prosaposin) of the ECM induces differentiation of MSCs to CAFs, enhanced proliferation, and survival of tumor cells
37	Adipose tissue	Human, rat	Gastric cancer	Promoting	MAPK activation, decrease apoptosis
38	Adipose tissue	Human	Leukemia	Suppressive	DKK‐1‐mediated inhibition
39	Bone marrow	Human	Colorectal cancer	Promoting	CCR5
40	Tumor tissue	Human	Colorectal cancer	Promoting	Tumor cells escape from senescence via P53/P21 pathway
41	Adipose tissue	Human	Lung cancer	Promoting	IL‐6/STAT3
42	Bone marrow	Human, mouse	Breast cancer	Promoting	Chemoresistance via a CD9‐dependent mechanism
43	Bone marrow, tumor tissue	Human, mouse	Prostate cancer	Promoting	Asporin (ASPN) secreted by MSCs drives metastasis

Within the tumor, MSCs can exert both stimulatory and inhibitory effects on cancer cell growth, invasion, and metastasis through direct or indirect interactions with tumor cells. However, despite the seemingly opposing potential effects of MSCs on tumor growth, their net effect seems to be predominantly pro‐tumorigenic. This reflects the imbalance between pro‐ and anti‐tumorigenic activities that may vary depending on tumor type (and regionally within the tumors), the ecology of the host milieu, the stage of the evolution of a particular tumor, and possibly the composition of the MSC population itself. The predominantly pro‐tumorigenic effect of MSCs *in vivo* and the opposing effects reported may be due to differences in experimental design, models used, and MSC heterogeneity that may reflect variable responses to a given set of stimuli.

For a complete list of abbreviations see supplementary material, Table S1.

## MSCs: heterogeneous cells in search of better definition

**Figure 1:**
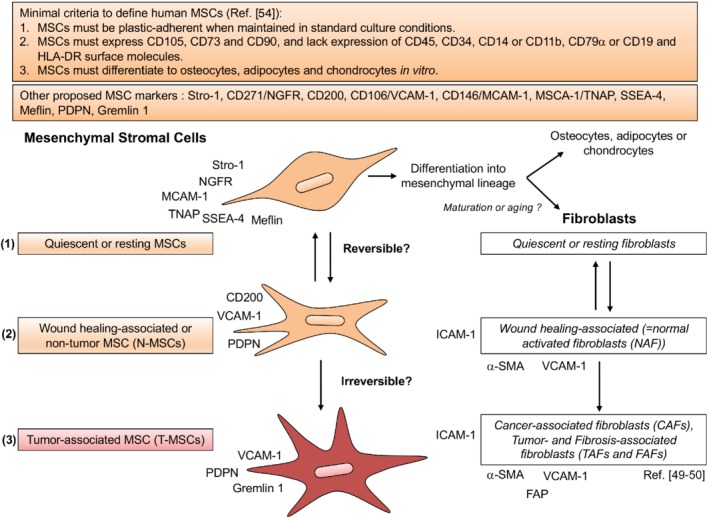
MSC definition and differentiation and comparison with fibroblasts. MSCs have been suggested to be a probable source of fibroblasts, implying that fibroblasts are one type of mesenchymal cell into which MSCs differentiate. However, as MSCs and fibroblasts share numerous functional features, it is possible that maturation or aging (although not in the sense of cell senescence) rather differentiation distinguish the two cell types. Fibroblasts may thus be a more ‘mature’ form of MSCs that have lost pluripotency and altered part of their cell surface receptor repertoire but that can respond to environmental stimuli such as injury and tumor growth in a manner akin to that of MSCs, many of whose properties they retain. MSC (left) and fibroblast (right) activation are illustrated under reversible, wound healing‐associated, and chronic tumor‐related inflammation. Some of the markers associated with each cell type in the context of wound healing and the tumor microenvironment are highlighted. (1) MSCs are a diverse and heterogeneous subset of multipotent precursors present in the stromal fraction of many adult tissues, especially bone marrow but also adipose tissue, synovial membranes, tooth pulp, and the connective tissue of most organs. Several studies show that MSCs lie adjacent to blood vessels and are localized in almost every perivascular space of the body. MSCs are the common predecessors of cells of the mesenchymal lineage, such as bone, cartilage, and fat cells. They can also differentiate into cells from unrelated germline lineages (endodermic and neuroectodermic differentiation potential), a process known as transdifferentiation. Quiescent or resting MSCs are spindle‐shaped cells (fibroblast‐like cells), but contrary to fibroblasts, which can be identified primarily based on morphology, MSCs are more heterogeneous. (2) In response to tissue injury and the associated stimuli, quiescent MSCs are activated to facilitate repair and regeneration. These MSCs may be tissue‐resident or recruited from the bone marrow or adjacent tissues and adopt a stellate morphology. The acquired synthetic properties are associated with secretory and migratory functions that amplify their activation, recruitment, and proliferation. Such activation may be reversed by reprogramming; alternatively, activated MSCs may undergo apoptosis upon completion of the repair process. (3) Chronic inflammation and/or the presence of a tumor induces prolonged activation of MSCs (tumor‐associated MSCs, T‐MSCs), which may gain further secretory properties (e.g. high secretion of IL‐6), specialized ECM remodeling ability, and robust autocrine activation and dynamic immunomodulatory signaling functions. Epigenetic regulation may help to maintain such activated states. T‐MSCs gain enhanced proliferative properties and their functional diversity adds to the dynamic complexity of the evolving tumor microenvironment. For a complete list of abbreviations see supplementary material, Table S1.

Precise definition of stromal cell populations is still lacking. Unlike hematopoietic cell subpopulations, whose identity, developmental stage, and plasticity can be predicted based on a combination of cell surface marker and transcription factor expression 45, 46, 47, stromal cells lack comparable functional and differentiation state markers. As a result, stromal cell populations are defined based on relatively loose phenotypic and functional criteria, which may be common to cells with distinct identities. Fibroblasts illustrate this notion well. Although a few cell surface receptors, including FAP (fibroblast activation protein α) and FSP (fibroblast surface protein), are commonly used to identify fibroblasts 48, 49, 50, their expression allows only approximate categorization of a subset of stromal cells. Moreover, fibroblasts are primarily defined based on their functional properties upon activation, during which they express alpha smooth muscle actin (α‐SMA) and secrete a wide range of extracellular matrix (ECM) components. These secretory products are more or less comparable in the context of wound healing (where the cells are labeled myofibroblasts) 51, 52 and cancer growth [where they are commonly referred to as cancer‐associated fibroblasts (CAFs)] 49, 50. Resting fibroblasts, which are identified largely based on morphology, remain poorly defined in terms of biological properties. Arguments have been put forth that they are multipotent cells, capable of differentiating into a spectrum of mesenchymal tissues 49, which is akin to tissue MSCs. However, adult skin fibroblasts tend not to differentiate into various mesenchymal tissues in culture and neither their origin nor their potential heterogeneity has been clearly elucidated 49, 53. Similar issues face the definition of MSCs (Figure 1).

Mesenchymal stem or stromal cells are multipotent stromal cells, believed to be an important constituent of the connective tissue that forms the supportive structure in which functional cells of tissues reside. In 2006, the International Society for Cellular Therapy (ISCT) published a position paper to define MSCs and recommended renaming the cells ‘multipotent mesenchymal stromal cells’ 54, 55. However, the most commonly used terms are ‘mesenchymal stromal cells’ and ‘mesenchymal stem cells’.

Although they were initially described in the bone marrow (BM), MSCs display a broad tissue distribution, including adipose, synovial, and lung tissue as well as umbilical cord and peripheral blood 56. Their defining properties include adhesiveness, a cell surface receptor repertoire, and plasticity. Adhesiveness is determined by MSC attachment to plastic under standard culture conditions; the cell surface phenotype is defined by expression of CD105, CD73, and CD90, and lack of expression of CD45, CD34, CD14 or CD11b, CD79α, CD19, and HLA‐DR. This includes low expression levels of MHC class I molecules, and minimal or no expression of MHC class II molecules or co‐stimulatory receptors, including CD40, CD80, and CD86, precluding antigen‐presenting activity 57. Plasticity is measured by the ability of the cells to differentiate into osteocytes, adipocytes, and chondrocytes *in vitro* in response to appropriate growth factors 54. However, MSCs have the capacity to differentiate into both mesodermal and non‐mesodermal tissues, such that in addition to osteocytes, adipocytes, and chondrocytes, they can differentiate toward endodermal and neuroectodermal lineages (multi‐lineage plasticity 56). Furthermore, a population of MSCs that displays homogeneous expression of CD105, CD90, and CD73 may display heterogeneous differentiation properties. Exposure to differentiation factors may result in only a fraction of the cells differentiating into adipocytes, chondrocytes or osteocytes, suggesting functional heterogeneity despite common cell surface marker expression. Whether such functional heterogeneity reflects differences in adaptation to *in vitro* culture or the outgrowth of stromal cell subpopulations from progenitor cells bearing distinct identities remains to be resolved. It must also be noted that the above criteria have been defined using bone marrow‐derived MSCs but that there are substantial phenotypic and functional differences among MSCs from different tissues 58.

Refinement of MSC definition along with the identification of their putative subpopulations requires additional phenotypic and/or functional criteria. Candidate markers that are associated with MSCs from various tissues include Stro‐1 (BM‐MSCs) 59, CD271/NGFR (nerve growth factor receptor) 60, CD200 61, CD106/VCAM‐1 (vascular cell adhesion molecule 1) 62, CD146/MCAM‐1 (melanoma cell adhesion molecule 1) 63, 64, MSCA‐1/TNAP (mesenchymal stromal cell antigen 1/tissue‐nonspecific alkaline phosphatase) 65, and SSEA‐4 (stage‐specific embryonic antigen 4) 66. The reliability and limitations of the most commonly used markers have recently been reviewed 67. Several newly identified promising candidate markers include Meflin 68, PDPN (podoplanin) 69, and gremlin‐1 70, 71, 72.

## Physiological role: tissue regeneration and wound repair

MSCs are widely believed to play a central role in tissue repair. Injury‐initiated inflammation, whose effectors include innate immune cells and their mediators, and the ensuing tissue remodeling provide signals that mobilize MSCs and drive their differentiation toward diverse stromal components, some of which replace damaged cells. Mesenchymal stromal cells may be injured‐tissue‐resident or recruited from the bone marrow. However, the mechanisms by which they are mobilized and recruited to damaged sites are not fully understood. One study, using a murine model of acute renal tubular necrosis, suggested that bone marrow‐derived MSC recruitment to sites of injury relies on the interaction between CD44 expressed on the MSC surface and hyaluronic acid produced by a variety of cells in areas of tissue remodeling 73. However, additional factors are likely implicated and the mechanisms that promote MSC survival and differentiation toward distinct cell types *in vivo* are still unclear 74.

The secretome and proteome of MSCs reflect their pleiotropic functions and plasticity 75. In response to soluble mediators derived from the microenvironment of injured tissues, including tumor necrosis factor*‐*alpha (TNF‐α), interleukin‐1 (IL‐1), interferon‐gamma (IFN‐γ), and toxins from infectious agents, MSCs can release a wide repertoire of soluble mediators that includes epidermal growth factor (EGF), fibroblast growth factor (FGF), platelet‐derived growth factor (PDGF), transforming growth factor‐beta (TGF‐β), vascular endothelial growth factor (VEGF), hepatocyte growth factor (HGF), insulin‐like growth factor‐1 (IGF‐1), angiopoietin‐1 (Ang‐1), keratinocyte growth factor (KGF), TNF‐stimulated G6 protein (TSG‐6), interleukin‐1 receptor antagonist (IL‐1RA), prostaglandin E2 (PGE2), indoleamine 2,3 dioxygenase (IDO), nitric oxide (NO), and stromal‐derived factor 1 (SDF‐1). These mediators promote the activation of fibroblasts, endothelial cells, and tissue progenitor cells, leading to angiogenesis, inhibition of apoptosis, ECM deposition, and damaged cell replacement 6, 76, 77, 78, 79, which in turn ensure tissue regeneration and repair 80, 81, 82. In addition to helping orchestrate regeneration and repair, MSCs can actively participate in bactericidal activity (through LL‐37) 83.

Several *in vivo* studies have shown the beneficial effect of allogeneic or xenogenic MSCs in a variety of disorders that require tissue regeneration and repair, including acute graft‐versus‐host disease, sepsis, acute asthma, acute renal failure, multiple sclerosis, and myocardial infarction 76, 84, 85, 86. Currently, more than 785 studies are underway or have been submitted to http://clinicaltrials.gov (https://clinicaltrials.gov/) under the terms ‘mesenchymal stem cells’ or ‘mesenchymal stromal cells’.

## MSCs and cancer cell crosstalk

**Figure 2:**
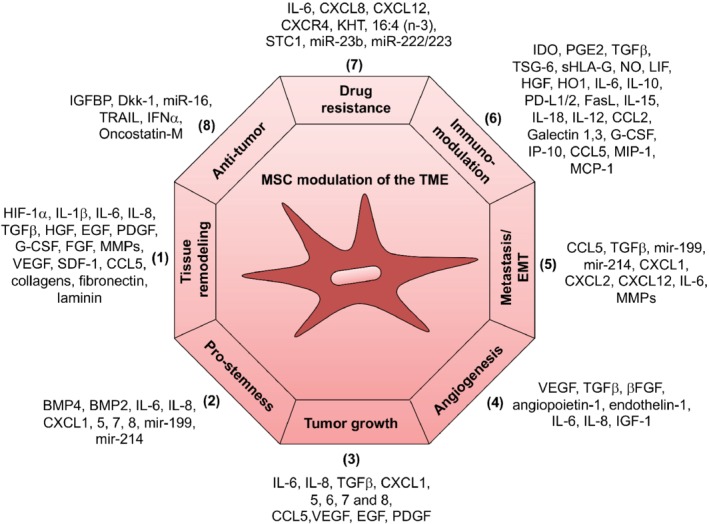
Summary of MSC effects on the tumor microenvironment. MSCs have multiple effects on tumor cells, mainly promoting tumor growth due, at least in part, to their role in regulating inflammation and tissue repair (1). They affect tumor cell survival and stemness (2 and 3) and contribute to tumor vasculature by producing angiogenic factors and by differentiating into pericytes (4). MSCs promote tumor cell motility, EMT, and metastasis, and secrete chemokines, including CXCL1, CXCL2, and CXCL12, and cytokines, including IL‐6 and several matrix metalloproteinases (MMPs), which degrade the ECM and facilitate tumor cell migration (5). They exert an important immunomodulatory function, which is primarily immunosuppressive (6) and can enhance tumor cell resistance to drugs, at least in part by releasing exosomes, which harbor numerous mediators, including miRNAs that may alter tumor cell properties (7). Although MSCs are primarily pro‐tumorigenic, several studies have shown that they may display anti‐tumor effects (8) as well. For a complete list of abbreviations see supplementary material, Table S1.

Accumulating evidence suggests that MSCs have the ability to migrate toward tumor sites 87 and MSC mobilization has been observed in response to a wide range of solid cancer‐derived cell types. Within the tumor microenvironment (TME), MSCs can exert both stimulatory and inhibitory effects on cancer cell growth, invasion, and metastasis through direct or indirect interactions with tumor cells (Table 1 and Figure 2). However, their net effect seems to be predominantly pro‐tumorigenic, which may reflect an imbalance between pro‐ and anti‐tumorigenic activity dictated by the tumor type, intratumoral heterogeneity, the ecology of the host milieu, and possibly the composition of the MSC population itself.

MSCs interact with and may affect the function of cancer cells at multiple stages of cancer progression. Within the primary tumor, MSCs have been observed to drive tumor cells toward acquiring invasive and metastatic properties. MSCs induce expression of epithelial–mesenchymal transition (EMT)‐ and hypoxia‐related genes in primary tumor cells and promote tumor cell dissemination 70. They also deposit ECM 88; participate in the remodeling of the TME; secrete IL‐6 and TGF‐β, which induces EMT; and help to create a niche that promotes angiogenesis and tumor invasion 5, 89. These observations are consistent with the predominantly pro‐tumorigenic effect of MSCs *in vivo*. The tumor inhibitory effects that have been reported may be due to differences in experimental design, models used, and MSC heterogeneity, which may reflect variable responses to a given set of stimuli (explored more extensively in a review by Klopp *et al*) 6, 90.

### Effect of the tumor microenvironment on the MSC phenotype

Tumor‐derived signals have the capacity to modulate the phenotype of tissue‐resident and tumor‐recruited MSCs (T‐MSCs), which become constituents of the tumor mass and harbor features distinct from those of normal tissue MSCs (N‐MSCs) or bone marrow MSCs (BM‐MSCs) 91. Differences between non‐tumor‐associated MSCs and T‐MSCs may arise in large part in response to cytokines and exosomes produced by the tumor microenvironment. This notion is supported by observations that MSCs treated with IFN‐γ and TNF‐α upregulate TGF‐β and VEGF expression 92, 93. TGF‐β can then promote EMT, which may facilitate invasion and metastasis 92. In addition, IFN‐γ and TNF‐α can enhance the immunosuppressive effects of MSCs 94, further helping tumor cell dissemination. Exosomes derived from breast and ovarian cancer cells can cause adipose tissue MSCs to adopt a CAF phenotype, characterized, in part, by upregulated α‐SMA expression, and can also promote MSC expression of SDF‐1, VEGF, CCL5 (RANTES), and TGF‐β 95, 96. Recently, Raz *et al* 97 have shown in breast tumors that resident and BM‐derived MSCs differentiate toward a subpopulation of CAF‐like cells that express distinct immune‐response‐related genes. Analysis of gene expression in these resident and BM‐derived CAF‐like cells from mammary tumors or their lung metastases revealed tissue‐specific transcriptional changes, implicating a microenvironmental influence on the reprogramming of stromal cells. Interestingly, BM‐derived CAF‐like cells were shown to be functionally important for tumor growth and were more efficient than their resident counterparts in promoting angiogenesis. Thus, MSCs recruited to neoplastic tissues can be reprogrammed in a local, tissue‐specific manner to induce tumor‐promoting inflammation and to facilitate angiogenesis and tumor growth 97.

### Characteristics of tumor‐associated MSCs (T‐MSCs)

Tumor‐associated MSCs do not undergo transformation and are euploid 98, 99. Moreover, MSCs are more prevalent in tumor tissues than in adjacent normal tissues 33, 70 and exhibit a significantly greater proliferative capacity than their normal tissue‐associated counterparts 99, 100, 101, 102. In addition, T‐MSCs exhibit a stronger migratory capability than N‐MSCs and more potent immunosuppressive activity than BM‐MSCs 99, 101, 102, 103, 104, 105, 106, 107. Finally, T‐MSCs have been shown to promote tumor cell proliferation 99 and to increase the proportion of cancer stem cells 99, 108, suggesting a possible role in tumor cell reprogramming.

## Pro‐inflammatory and immunosuppressive effects of MSCs in the TME: Dr Jekyll and Mr Hyde behavior?

As discussed above, MSCs affect the immune response by secreting immunomodulatory molecules as well as by cell–cell contact. Several studies have also highlighted the role of exosomes and other extracellular vesicles (EVs) on MSC‐mediated immune modulation 109, 110. Two major functional features of MSCs that are relevant to immunity include their ability to induce immunosuppression and to exert immunoprivilege. Immunosuppression has primarily been associated with BM‐MSCs, whereas studies using other sources of MSCs have shown both immunosuppressive and pro‐inflammatory effects. These seemingly contradictory observations may be due to species‐specific factors but possibly also to the tissue from which the MSCs were harvested and to priming by their microenvironment. The mechanisms underlying immunoprivilege are largely unknown but are most probably related to low expression of MHC I and MHC II molecules coupled to the immunosuppressive functions of MSCs. Furthermore, immunoprivilege is not a stable property: cellular differentiation and priming by IFN‐γ upregulate MHC‐I and, to a lesser extent, MHC‐II expression, enhancing MSC antigen‐presenting capacity and immunogenicity and reducing immunoprivilege 111.

**Figure 3:**
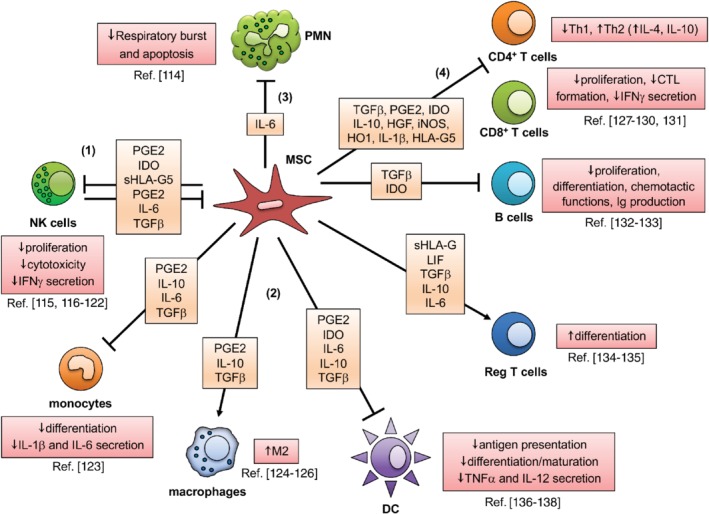
MSC and immune cell interactions. (1) MSCs can inhibit the proliferation, cytotoxicity, and cytokine production by NK cells by secreting several mediators, including PGE2, IDO, and sHLA‐G5. In turn, MSCs can be killed by cytokine‐activated NK cells through the engagement of NKG2D by its ligand ULBP3 or MICA expressed by MSCs, and of DNAM‐1 by MSC‐associated PVR or nectin‐2. (2) MSCs inhibit differentiation of monocytes to DCs, skew mature DCs toward an immature DC state, and inhibit TNF‐α and IL‐12 production by DCs through PGE2 secretion. (3) MSCs dampen the respiratory burst and delay spontaneous apoptosis of neutrophils by constitutively releasing IL‐6. (4) MSCs affect CD4^+^ T cells through PGE2, IDO, TGF‐β, HGF, iNOS, and HO1 release. MSCs increase the production of IL‐4 and IL‐10 by Th2 cells and reduce the release of IFN‐γ by Th1 and NK cells. IDO can reduce tryptophan levels and inhibit the growth of B cells, T cells, and NK cells. Defective CD4^+^ T‐cell activation impairs helper function for B‐cell proliferation and antibody production. CD8^+^ T‐cell cytotoxicity is inhibited mainly by sHLA‐G5, as well as by the increase of the regulatory T‐cell population, also induced by IL‐10. Adapted from Refs. 9, 112, 116. For a complete list of abbreviations see supplementary material, Table S1.

The immunosuppressive effects of MSCs require proximity to their target cells, which include T and B lymphocytes as well as NK cells (Figure 3). Activated/primed MSCs upregulate MHC class I molecules, ICAM‐1 and VCAM‐1 adhesion receptors, and the immunosuppressive molecule PD‐L1 (programmed death‐ligand 1). The latter three molecules recognize ligands on immune cells, promote cell–cell adhesion, and facilitate immune cell exposure to secreted immunosuppressive mediators 139. Following activation, the MSC‐derived secreted immunosuppressive arsenal includes HLA‐G, TGF‐β, PGE2, TSG‐6 (tumor necrosis factor‐inducible gene 6 protein), HO‐1 (heme oxygenase 1), HGF, IL‐10, IL‐6, IDO1 (indoleamine‐pyrrole 2,3‐dioxygenase), ARG1/2 (arginase), NOS2 (nitric oxide synthase 2A), adenosine, and LIF (leukemia inhibitory factor), as well as PD‐L1/2 and Fas ligand (FasL) 140. TGF‐β and PGE2 are two key mediators of immunosuppression. TGF‐β directly inhibits the function of anti‐tumor effector cells (NK, CD8^+^ T cells, and γδ T cells) by downregulating the activating receptor NKG2D and generating and recruiting regulatory T cells and γδ T cells 4, 112, 143, 144, 145, 146, 147, 148, 149, 150.

### MSCs and innate immunity

MSCs exert their pro‐ and anti‐inflammatory effects by a variety of mechanisms. They have a functional relationship with the complement system, as BM‐MSCs express the anaphylatoxin receptors C3aR and C5aR, suggesting that C3a and C5a may be chemotactic for MSCs toward sites of injury. MSCs also express the complement inhibitors CD46, CD55, and most predominantly CD59, which protect them from complement opsonization and lysis and secrete the complement inhibitor, factor H 144, 152, 153.

MSCs play an active role in neutrophil recruitment by secreting chemotactic cytokines and chemokines, including IL‐6, IL‐8, IFN‐β, GM‐CSF, and macrophage inhibitory factor (MIF). They also promote neutrophil survival, which helps to eliminate pathogens 114 and to respond to histamine released by mast cells by inducing IL‐6 production 117.

MSCs directly and indirectly interfere with the proliferation, cytokine production, and, in some cases, cytotoxicity of NK cells. MSC–NK cell interactions are complex and largely dependent on the microenvironment and activation status of the NK cells. Bone marrow‐derived MSCs can inhibit NK cell proliferation, cytotoxicity, and cytokine production by secreting IDO1, TGF‐β, HLA‐G, and PGE2 103, 116, 117, 118, 119, 120. However, they are also vulnerable to lysis by activated NK cells, depending on their expression of activating NK receptor ligands, including the MHC class I polypeptide‐related sequence (MICA, B), UL16 binding proteins (ULBPs), CD112, and CD155 120, 121, 122. In human lung cancers, T‐MSCs have been shown to be more immunosuppressive than N‐MSCs, mainly through PGE2 and, to a lesser extent, IL‐6 secretion. They decrease IFN‐γ production and downregulate expression of the activating NK cell receptors NKp44, NKp30, NKG2D, DNAM‐I, and NKG2A. T‐MSCs also induce an inversion in the CD56^bright/dim^ NK cell ratio in favor of the CD56^dim^ phenotype, which is associated with degranulation rather than elevated cytokine production, in a contact‐dependent manner 103.

Dendritic cell (DC) function is affected by MSCs, which can directly inhibit both the maturation of monocytes and CD34^+^ precursor cells toward DCs as well as activation of DCs via PGE2, IL‐6, TSG‐6, and M‐CSF (macrophage colony‐stimulating factor) secretion, and Jagged‐2 mediated signaling 136, 137, 138. Both immature and mature DCs (iDCs and mDCs) are affected by MSCs. In the presence of MSCs, iDCs display diminished capacity to present antigen and stimulate T‐cell proliferation and naïve T‐cell differentiation, resulting in ineffective T‐cell activation. MSCs can also revert mDCs to an immature phenotype associated with downregulation of their cell surface expression of antigen‐presenting and co‐stimulatory molecules, suppression of IL‐12 secretion, and the inability to stimulate lymphocyte proliferation *in vitro* 138.

MSCs seem to favor the emergence of the myeloid suppressor cell (MDSC) phenotype by secreting IL‐6, HGF, and CXCL3 (C‐X‐C motif chemokine ligand 3), which stimulate MDSC production of COX2 (cyclooxygenase‐II enzyme, PTGS2), IDO, PD‐L1, and PD‐L2, and MMP9 (matrix metalloproteinase 9) 156, 157.

Mesenchymal stromal cells direct monocyte mobilization from the BM and macrophage recruitment to sites of inflammation to promote wound repair through secretion of the chemokine (C‐C motif) ligands CCL2, CCL3, and CCL12. They also participate in the differentiation of monocytes to M2 (tissue repair‐associated) macrophages via direct cell contact and secretion of PGE2, IL‐6, and IDO 124, 125. The capacity of MSCs to regulate the macrophage phenotype (M1 or M2) and to promote immunosuppression strongly depends on macrophage IL‐6 signaling 126. Finally, as discussed above, MSCs impair the maturation and differentiation of antigen‐presenting cells (APCs) 123.

Current research is exploring the effect of MSCs on γδ T cells. γδ T cells have the ability to produce the pro‐inflammatory cytokines IFN‐γ, TNF‐α, and IL‐17, as well as the anti‐inflammatory cytokines TGF‐β, IL‐4, and IL‐10, depending on the types of signals that predominate in the tissue microenvironment, and exert both anti‐ and pro‐tumoral effects 158. TGF‐β acts as a key player in the MSC‐mediated regulatory response by inducing CD4^+^ regulatory T cells (Treg) and γδ regulatory T cells. On the other hand, MSCs are potent suppressors of γδ‐cell proliferation, cytokine production, and cytolytic responses (anti‐tumor effect) *in vitro* through COX2‐dependent production of PGE2 134.

### MSCs and adaptive immunity

MSCs can regulate the activation and function of T and B lymphocytes 9, 113. Many factors have been reported to be critical in MSC‐mediated suppression of T‐cell proliferation, including iNOS (inducible NO synthase), IDO, semaphorin‐3A, B7‐H4, HLA‐G, LIF, galectin(s), HO‐1, IL‐6, IL‐10, PD‐L1/2, FasL, and PGE2 127, 128, 129. MSCs exert inhibitory effects toward Th1 and Th17 (pro‐inflammatory) cells through PD‐1, PGE2, and IL‐10, and promotion of Th2 secretion of IL‐4 130. However, stimulatory effects on Th17 cells have also been observed. MSCs can promote Treg differentiation by secreting TGF‐β, IL‐6, and IL‐10, and expressing IDO 135.

The immunosuppressive function of MSCs is elicited by IFN‐γ, which induces the production of chemokines, IDO, PGE2, HGF, and TGF‐β in humans to attract and to suppress T cells 131, 159. Although soluble factors are critical to mediate the immunosuppressive functions of MSCs, cell–cell contact is involved in MSC‐based immunosuppression of T cells, including ICAM‐1–LFA‐1 and PD‐1/PD‐L1 interactions. Additional mechanisms of suppression occur through microRNA and exosome release 159, 160, 161.

B‐cell–MSC interactions are less well studied, although observations suggest an inhibitory effect on B‐cell activation, proliferation, differentiation, and chemotactic responses 132, 133.

### Priming: Toll‐like receptors and the level of inflammation

**Figure 4:**
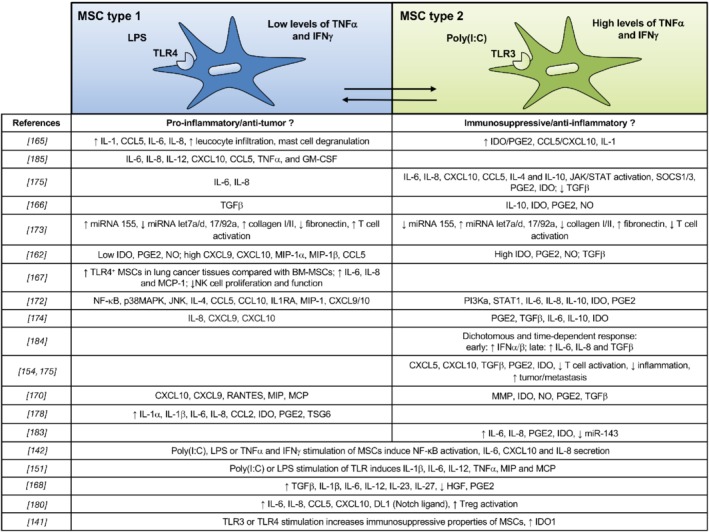
The MSC polarization paradigm. The level of inflammation and/or TLR agonists polarizes MSCs toward a pro‐inflammatory/anti‐tumor (MSC1) or an anti‐inflammatory/immunosuppressive/pro‐tumor (MSC2) phenotype. Low levels of TNF‐α, IFN‐γ (low level of inflammation), and/or TLR4 agonists (LPS) polarize MSCs toward a pro‐inflammatory phenotype, whereas the downstream consequences of high TNF‐α/IFN‐γ/TLR3 [poly(I:C)] stimulation skew MSCs toward an anti‐inflammatory MSC2 phenotype. MSC1 and MSC2 have divergent cytokine and chemokine secretion repertoires, differences in differentiation capability, extracellular matrix deposition, TGF‐β signaling pathways, and Jagged, IDO, and PGE2 expression. The most compelling outcome is the opposite effect of the two cell types on immunomodulation. As shown in the figure, existing data are conflicting regarding the repertoire of cytokines and chemokines secreted in response to the numerous stimuli from the microenvironment. MSCs are a heterogeneous cell population, and the range of the observed responses may be explained, at least in part, by their diversity itself. For a complete list of abbreviations see supplementary material, Table S1.

**Figure 5:**
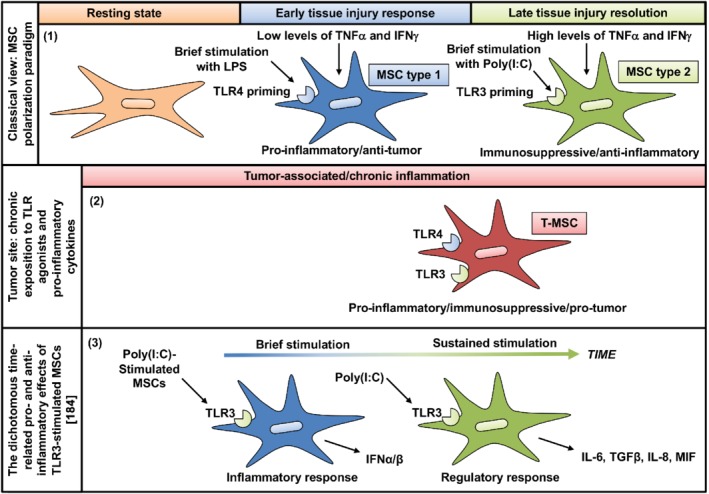
TLR priming of MSCs. (1) The classical view of MSC polarization: different levels of inflammation and/or TLR agonist stimulation polarize MSCs toward a pro‐inflammatory/anti‐tumor (MSC1) or anti‐inflammatory/immunosuppressive/pro‐tumor (MSC2) phenotype. Low levels of TNF‐α, IFN‐γ (low level of inflammation), and/or TLR4 agonists polarize MSCs toward a pro‐inflammatory phenotype, whereas stimulation with high TNF‐α/IFN‐γ/TLR3 levels promotes induction of an anti‐inflammatory MSC2 phenotype. (2) In the tumor microenvironment, MSCs are continuously exposed to pro‐inflammatory cytokines and tend to express both TLR3 and TLR4. They acquire features that help to sustain tumor progression and that overlap with those defined by the classical MSC1 and MSC2 phenotypes. (3) Petri *et al* showed that the dichotomous pro‐ and anti‐inflammatory effects of TLR3‐stimulated MSCs may be time‐related 184. MSCs may initially respond by secreting pro‐inflammatory IFN‐α/β and later switch to production of the regulatory factors IL‐6 and TGF‐β. MSCs may thereby actively contribute to each phase of wound healing, progressively driving the process to completion and restoration of tissue function. For a complete list of abbreviations see supplementary material, Table S1.

MSC immunomodulatory activity is reported to be primed by cytokines from a pro‐inflammatory microenvironment (for example, inflammation, cancer or infection), particularly IFN‐γ, TNF‐α, and IL‐1β, and by Toll‐like receptor (TLR) stimulation 162, 163, 164, 165, 166, 167, 168. MSCs express *TLR1–6* transcripts and TLR2–4, 7, and 9 proteins, and can be polarized toward a pro‐inflammatory or an immunosuppressive phenotype following specific TLR stimulation 169, 170, 171, 172. Thus, TLR4‐primed MSCs are polarized toward a pro‐inflammatory MSC1 phenotype (tumor‐growth inhibition), whereas TLR3‐primed MSCs are polarized toward the more classical immunosuppressive MSC2 phenotype (Figure 4) 173, 175. The degree of inflammation, as assessed by the cytokine repertoire and production level within the microenvironment, appears to be critical in MSC polarization. MSCs become immunosuppressive only when exposed to high levels of pro‐inflammatory cytokines (TNF‐α, IFN‐γ), which corresponds to late stages of inflammation 139, 176, but the MSC2 phenotype is also influenced by TLR3 stimulation 176. In the presence of low levels of TNF‐α and IFN‐γ, at the early phase of inflammation, MSCs may adopt a pro‐inflammatory phenotype (MSC1) and enhance immune responses, in part through TLR4 expressed on their surface 175. TLR4 stimulation promotes IL‐6, IL‐8, and TGF‐β secretion, whereas TLR3 stimulation increases IL‐4, IL‐1RA (interleukin‐1 receptor antagonist), IDO, and PGE2 164, 175, 177, 178. However, results are discordant among research groups and MSC activation through TLR3 and 4 is also reported to lead to the secretion of IL‐1, IL‐6, IL‐8, TRAIL (TNF‐related apoptosis‐inducing ligand), and CCL5 174, 175, 179. Some studies have shown that both TLR3 and 4 enhance immunosuppression through IDO 141, whereas others have reported an increase in pro‐inflammatory cytokines in both 142, 151, 154, 179, 180. Thus, MSC licensing to become activated depends on stimulation by pro‐inflammatory cytokines, priming signals delivered by TLRs, and the timing of MSC engagement in immune effector cell activation (Figure 5) 164.

Experimentally, the duration of TLR stimulation of MSCs seems to play a role in their subsequent activation profile. Brief stimulation of MSCs with poly(I:C) led to an MSC2, whereas LPS stimulation induced an inflammatory MSC1 phenotype 175. However, MSCs at an infection site are likely to be continuously exposed to TLR agonists.

In contrast to the study of Waterman *et al* 175, other investigators have shown that TLR3 stimulation of MSCs leads to a pro‐inflammatory response 181, 182, 183, 184. The dichotomous pro‐ and anti‐inflammatory effects of TLR3‐stimulated MSCs may be time‐related. Thus, upon arriving at an infection site, MSCs may initially respond by secreting pro‐inflammatory IFN‐α/β and later switch to production of the regulatory factors IL‐6 and TGF‐β (Figure 5). They observed that the effect of constitutively produced TGF‐β was modulated by the presence of inducible IL‐6. MSCs may thereby actively contribute to each phase of wound healing, progressively driving the process to completion and restoration of tissue function 184.

Several mediators, including COX1, COX2, LIF, HGF, Gal‐1, HO‐1, IL‐11, IL‐8, IL‐6, and TGF‐β, were observed to display variable constitutive expression in BM‐MSCs. Inflammatory priming of the cells differentially modulated expression of these mediators, strongly increasing expression of COX2, LIF, HGF, IL‐11, IL‐8, and IL‐6, while decreasing that of COX1, Gal‐1, and TGF‐β 185. According to one model 186, strong inflammation, characterized by IFN‐γ, TNF‐α, IL‐1, and IL‐17 production, induces NO/IDO production by MSCs, leading to an immunosuppressive phenotype, with secretion of TGF‐β, sHLA‐G, IL‐6, IL‐10, and IDO. In contrast, MSCs subjected to a microenvironment enriched in TGF‐β and IL‐10 acquire a pro‐inflammatory phenotype. Interestingly, MSCs themselves produce abundant TGF‐β, which can act in an autocrine manner to modulate their immunoregulatory functions 187. Thus, TGF‐β can reverse the immunosuppressive effect of MSCs induced by IFN‐γ and TNF‐α 186.

## MSC‐derived exosomes

Mesenchymal stromal cell‐derived extracellular vesicles (MSC‐EVs) play a significant role in the TME 188, 189. Release of EVs is a mechanism of intercellular communication used by tumor cells and stromal cells within the TME. EVs include exosomes, the smallest EV fraction arising from intracellular endosomes, and microvesicles generated by budding from the plasma membrane. EVs can directly activate target cell surface receptors through protein and bioactive lipid ligands, and by delivering different effectors, including transcription factors, oncogenes, proteins, growth factors, and non‐coding regulatory RNAs, thus inducing functional changes in recipient cells.

### MSC‐EVs are regulators of tumor cell survival and growth

Similar to their effects on immunity and the inflammatory response, MSCs have shown divergent effects on tumor cells, some of which are anti‐, whereas others are pro‐tumorigenic. Similar to MSCs, MSC‐EVs have a dual effect on tumor progression. Exosomes derived from MSCs in non‐small cell lung cancer have been shown to promote chemoresistance 190. Tumor‐EVs can also mediate drug resistance through mechanisms that include drug sequestration; delivery of specific mRNAs, miRNAs, and proteins; and crosstalk between cancer cells and MSCs 191, 192. MSC‐EVs have been found to modulate the tumor microenvironment, creating favorable conditions for cancer cell metastasis, and have been shown to mimic the effects of MSCs on tumor growth promotion 193. Kalluri 194 described tumor‐associated and circulating exosomes as heterogeneous populations that generate a unique tumor nanoenvironment (TNE).

### Immunomodulation

By fulfilling the role of vehicles that deliver immunomodulatory mediators, MSC‐EVs display functions similar to those of their parent cells 195. Cytokines (including IL‐6, IDO, PGE2, IL‐10) and chemokines (including CXCL2, CCL2, CXCL8) may be packaged into EVs together with nucleic acids and post‐transcriptional modulators which can influence the inflammatory response when released 195, 196. Exosomes can inhibit B‐cell proliferation 197 and increase Treg activity 198. In physiological conditions, MSC‐EVs have been reported to modulate the immune cell response to facilitate tissue repair, through promotion of anti‐inflammatory and pro‐regenerative (M2) macrophages over pro‐inflammatory M1 macrophages and concomitant enhancement of the expression of the anti‐inflammatory cytokines IL‐10 and TGF‐β 199. In tumors, chronic inflammation promotes immunosuppression, at least in part through EV release, which contributes to tumor progression 200, 201.

## Interleukin‐6

Aside from TGF‐β and PGE2, IL‐6 appears to play a major role in MSC communication with their microenvironment. IL‐6 is a pleiotropic cytokine, highly secreted by tumor stromal cells, including MSCs. The IL‐6 signaling pathway consists of IL‐6Rα (CD126) and gp130 (CD130), JAK/STAT signaling, and negative regulation by SOCS molecules. IL‐6 supports cancer cell proliferation, survival, and metastatic dissemination. Moreover, IL‐6 can act on numerous cell types within the tumor microenvironment to sustain a pro‐tumoral milieu by supporting angiogenesis and tumor evasion of immune surveillance. However, IL‐6 may also promote anti‐tumor adaptive immunity 202, 203.

MSCs, especially MSCs isolated from the tumor stroma, secrete higher levels of IL‐6 70, 103 than other non‐tumor cells, which together with PGE2 can participate in suppression of NK cell activity and facilitate tumor cell dissemination and metastasis 70. IL‐6 secretion by MSCs has been shown to be part of the late regulatory response 184, which includes TGF‐β, to induce senescent‐like NK cells. TGF‐β secreted by MSCs in osteosarcoma can increase the migratory capacity of tumor cells, which, in turn, stimulate the secretion of IL‐6 that fosters cancer cell stemness and aggressiveness 204, 205. In a model of arthritis, IL‐6‐dependent PGE2 secretion by MSCs inhibits local inflammation 206. Indeed, IL‐6 modifies the soluble mediator secretion profile of MSCs, increasing PGE2 and VEGF, among others. MSC‐derived IL‐6 and PGE2 skew monocyte differentiation toward the formation of IL‐10‐expressing macrophages 207. IL‐6 clearly plays an important role in the crosstalk between MSCs and the tumor microenvironment, and additional work is needed to elucidate the full spectrum of its effects. In different tumor models, targeted inhibition of IL‐6 may enhance the efficacy of anti‐PD‐L1 208, 209, 210.

## MSCs and anti‐tumor therapy

The potential therapeutic benefit of exogenous MSCs has been under preclinical investigation since the late 1990s. Tissue regeneration‐related candidate MSC applications include bone marrow transplantation, graft‐versus‐host disease (GVHD), acute myocardial infarction, heart failure, stroke, lung diseases, acute kidney failure, liver fibrosis, juvenile diabetes, osteoarthritis and rheumatoid arthritis, inflammatory bowel disease, multiple sclerosis, Parkinson's disease, and sepsis 211, 212, some of which are approved (GVHD being one example) 213, 214, 215. There are currently also ongoing clinical trials that use MSCs to treat tumors 7. In the context of such cell therapeutic approaches, MSCs are used as gene delivery vehicles for tumor‐targeted therapies 216, 217, 218. MSCs have been engineered to deliver interleukins to improve anti‐cancer immune surveillance, as delivery agents of interferons (IFN‐α and ‐β) 159, and as carriers of prodrugs or oncolytic viruses 219, 220. MSCs have also been tested as deliverers of anti‐angiogenic agents, pro‐apoptotic proteins (TRAIL, for example) 221, and growth factor antagonists. Moreover, after exposure to high doses of chemotherapeutic drugs such as paclitaxel or gemcitabine, MSCs have been shown to accumulate and deliver the anti‐neoplastic agent without undergoing genetic modifications and to decrease tumor growth 222, 223. Their ability to preferentially migrate toward tumor sites (primary and metastatic neoplasms) in addition to their availability, non‐immunogenic nature, and relative ease of manipulation *in vitro* renders them attractive candidates for cell‐based therapies. However, as discussed above, increasing evidence regarding the tumor‐promoting activity of MSCs, especially when subverted by the TME, raises issues as to their safety and cautions their use in clinical trials. In addition, using engineered MSCs may be a problem after eradication of the tumor they are designed to target 224. A recent systematic review by Christodoulou *et al* addresses these issues in the settings of preclinical cancer cytotherapy 225.

### MSC‐EVs for anti‐tumor therapy

Several studies suggest that the cell source may condition EV homing to specific sites and that their membrane can be engineered to increase tissue‐specific targeting 226, 227. These observations open new possibilities for potential future applications of MSC‐EVs as cell‐free therapeutic agents 228. MSC‐derived exosomes may thus be used as delivery vehicles to transfer genetic materials, including mRNA and non‐coding RNAs to recipient cells 160, 229, 230, 231, 232.

### Targeting the pro‐tumor effects of MSCs

Approaches that could be used to target MSCs in the TME and counteract their immunosuppressive effects include direct blockade of their immunosuppressive function and reprogramming to render their immunostimulatory properties dominant over their immunosuppressive ones 5. MSC activity could be modulated in a variety of ways using, among others, drugs that inhibit one or, preferably, several of the MSC‐derived immunosuppressive molecules (e.g. IDO, TGF‐β); inhibitory antibodies (e.g. anti‐PDGF, anti‐EGFR antibodies) that block the effect of growth factors involved in MSC–tumor cell crosstalk 233, 234, 235 and elicit ADCC (antibody‐dependent cellular cytotoxicity); inhibitors of sheddases/ADAMs (a disintegrin and metalloproteinases); and tyrosine kinase inhibitors. Some of these approaches have the advantage of targeting both MSCs and tumor cells. HMG‐CoA reductase inhibitors (statins) decrease mevalonate, its metabolic product that is essential for MSC and tumor cell metabolism. However, mevalonate is also required to develop an immune response and kill tumor cells. Thus, it is important to design inhibitors that can be directly and specifically delivered to MSCs 236.

PD‐L1 expression is upregulated in MSCs by IFN‐γ 237, and PD‐L1/PD1 is involved in MSC regulation of T‐ and B‐cell proliferation 238, 239. ADAM proteins release MSC ligands for NK cells and decrease NK‐cell recognition of tumor cells. ADAMs can be released in exosomes and microvesicles.

Reprogramming MSCs from an immunosuppressive to an immunostimulatory phenotype may constitute another potentially promising approach. The effects of bisphosphonates 240, 241, as well as immunomodulatory drugs such as thalidomide and lenalidomide, that target the TME and decrease IL‐6 by regulating SOCS1 242 are being assessed by several groups.

### Preclinical models

Studies on the effect of MSCs on cancer growth and their immunomodulatory properties have been based mainly on *in vitro* 2D co‐culture systems and on *in vivo* cancer models using primarily BM‐MSCs and adipose tissue‐isolated MSCs. New approaches using more complex *in vitro* 3D models are under development and are gaining interest, as they are more prone to mimic the *in vivo* features of the tumor microenvironment 204. For a more detailed review, the recent publication of Avnet *et al* 204 summarizes the different 3D preclinical models available, as well as their limitations.

## Concluding remarks

Mesenchymal stem cells may be heralded as key preservers of tissue homeostasis. Their pleiotropic nature provides them with the unique ability to act as sensors of tissue state and as both coordinators of and participants in the effector functions required to repair and regenerate injured tissues. By sensing the degree of an inflammatory response to injury, MSCs may adapt their own regulatory and effector functions to temper or boost the response as required. Thus, they can become immunosuppressive upon exposure to elevated levels of pro‐inflammatory cytokines while providing support to tissue repair by secreting ECM components and stimulating regeneration by resident stem cells. By contrast, in the presence of low levels of IFN‐γ and TNF‐α, MSCs may adopt a pro‐inflammatory phenotype and enhance T‐cell responses as well as tissue remodeling.

Despite its highly beneficial effects in the maintenance of homeostasis, MSC plasticity is a double‐edged sword as it can be readily exploited by tumors to serve tumor cell needs. In response to tumor‐derived cytokines and signals generated by direct physical contact with tumor cells, MSCs can adopt a potent immunosuppressive phenotype that acts on both innate and adaptive immunity. As a result, they may facilitate tumor progression, which entails acquisition by tumor cells of additional genetic and epigenetic changes that may shield them from cytotoxic cells and drugs and support their formation of metastases.

Although a plethora of studies have been conducted on MSCs in recent years, several key issues remain to be resolved. Perhaps the most pressing one is the heterogeneity of MSCs, which requires the identification and definition of their putative subpopulations and the determination of their mutual relationships. For example, are the different subpopulations stable or are they transient and can one subpopulation transform itself into another in response to microenvironmental stimuli? Several other questions need to be addressed as well. What is the relationship between MSCs and ‘resting’ fibroblasts – are they distinct entities or one and the same, perhaps at different stages of differentiation? What proportion of the stromal response to injury is directed by MSCs versus other more differentiated stromal cells? These and other issues will need to be resolved if we hope to effectively disrupt the functional support that MSCs provide to tumor growth.

## Author contributions statement

Both authors contributed to this review and reviewed the final manuscript.

## Supporting information


**Table S1.** Complete list of abbreviations used
