# Supplementary material for: Mesenchymal stromal cells in cancer: a review of their immunomodulatory functions and dual effects on tumor progression
Source: J Pathol. 2019 Dec 18;250(5):555–72. doi: 10.1002/path.5357 (PMC7217065; doi:10.1002/path.5357)
Supplement: Supplementary file 1 — Table S1. Complete list of abbreviations used [file PATH-250-555-s001.docx]

**Mesenchymal stromal cells in cancer: a review of their immunomodulatory functions and dual effects on tumor progression**

Galland and Stamenkovic. *J Pathol* DOI: 10.1002/path.5357

**Table S1.** Complete list of abbreviations used

**A**

*α-SMA: Alpha-smooth muscle actin*

*ANG1: Angiopoietin 1*

*AT: Adipose tissue*

**B**

*BM: Bone-marrow*

*BMPs: Bone morphogenetic proteins*

**C**

*CAF: Cancer-associated fibroblast*

*CCL: C-C motif chemokine ligand*

*CXCL: Chemokine (C-X-C motif) ligand*

*CXCR: C-X-C chemokine receptor*

**D**

*DCs: Dendritic cells*

*DKK-1: Dickkopf-related protein 1*

*DLL1: Delta-like canonical Notch ligand 1*

*DNAM1: DNAX accessory molecule-1*

**E**

*ECM: Extracellular matrix*

*ECs: Endothelial cells*

*EGF: Epidermal growth factor*

*EMT: Epithelial-to-mesenchymal transition*

**F**

*FAP: Fibroblast activation protein*

*FasL: Fas-ligand*

*FGF: Fibroblast growth factor*

**G**

*G-CSF: Granulocyte colony-stimulating factor*

*GM-CSF: Granulocyte–macrophage colony-stimulating factor*

**H**

*HGF: Hepatocyte growth factor*

*HIF: Hypoxia-inducible factor*

*HLA-DR: Human leukocyte antigen – DR isotype*

*HO1: Heme oxygenase*

**I**

*ICAM-1: Intercellular adhesion molecule 1*

*IDO: Indoleamine 2,3-dioxygenase*

*IFN: Interferon*

*IFN-γ: Interferon gamma*

*Ig: Immunoglobulin*

*IGF: Insulin-like growth factor-1*

*IGFs: Insulin‐like growth factors*

*IGFBP: insulin-like growth factor binding protein*

*IL: Interleukin*

*IL1RA: Interleukin-1 receptor antagonist*

*iNOS: Inducible NO synthase*

*IP-10: Interferon γ-induced protein 10 kDa (= CXCL10)*

**J**

*JAK: Janus kinase*

*JNK: c-Jun N-terminal kinase*

**L**

*LIF: Leukemia inhibitory factor*

*LPS: Lipopolysaccharides*

**M**

*MAPK: Mitogen-activated protein kinase*

*MCAM-1: Melanoma cell adhesion molecule*

*MCP-1: Monocyte chemoattractant protein 1 (= CCL2)*

*MICA: MHC class I polypeptide-related sequence A*

*MIF: Macrophage migration inhibitory factor*

*MIP-1: Macrophage inflammatory proteins (MIP-1α/β = CCL3/4)*

*miR: MicroRNA*

*MMP: Matrix metalloproteinase*

*MSC: Mesenchymal stromal cell*

**N**

*Nectin-2: Poliovirus receptor-related 2 (PVRL2)*

*NF-κB: Nuclear factor-kappa B*

*NGFR: Nerve growth factor receptor*

*NK: Natural killer cells*

*NKG2D: Natural-killer group 2, member D*

*NO: Nitric oxide*

**P**

*PARP: Poly(ADP-ribose) polymerase*

*PDGF: Platelet-derived growth factor*

*PDPN: Podoplanin*

*PD-L1/2: Programmed death-ligand 1*

*p-EGFR: Phosphorylated epidermal growth factor receptor*

*PGE2: Prostaglandin E2*

*PI3Ka: Phosphoinositide 3-kinase family, accessory domain*

*PMN: Polymorphonuclear leukocytes*

*Poly(I:C): Polyinosinic:polycytidylic acid*

*Polyunsaturated fatty acids such as KHT: 12-oxo-5,8,10-heptadecatrienoic acid and 16:4(n-3): hexadeca-4,7,10,13-tetraenoic acid*

*PVR: Poliovirus receptor*

**R**

*ROS: Reactive oxygen species*

**S**

*SDF1 (SDF-1): Stromal cell-derived factor 1 (= C-X-C motif chemokine ligand 12)*

*sHLA-G: Soluble human leukocyte antigen-G*

*sHLA-G5: Soluble human leukocyte antigen-G5*

*SOCS: Suppressor of cytokine signaling*

*SSEA4: Stage-specific embryonic antigen-4*

*STAT: Signal transducer and activator of transcription*

*STAT3: Signal transducer and activator of transcription 3*

*STC1: Stanniocalcin 1*

**T**

*TGF: Transforming growth factor*

*TGF-β: Transforming growth factor beta*

*TLRs: Toll-like receptors*

*TNF: Tumor necrosis factor*

*TNAP: Tissue-nonspecific alkaline phosphatase*

*TME: Tumor microenvironment*

*TRAIL: Tumor-necrosis-factor related apoptosis inducing ligand*

*Treg: Regulatory T cells*

*TSG6: TNF-stimulated gene 6 protein*

**U**

*UCB: Umbilical cord blood*

*ULBP3: UL16 binding protein*

**V**

*VCAM-1: Vascular adhesion molecule 1*

*VEGF: Vascular endothelial growth factor*

**X**

*XIAP: X-linked inhibitor of apoptosis protein*
